# Skill vs. Disposition: Examining Paths of Intervention Effects in an Alcohol and Drug Use Prevention Trial Targeting U.S. Adolescents

**DOI:** 10.1007/s10935-025-00881-8

**Published:** 2025-11-19

**Authors:** William B. Hansen, Ralph B. McNeal

**Affiliations:** 1 Prevention Strategies , Greensboro, NC USA; 2 Department of Sociology, University of Connecticut , Storrs, CT USA

**Keywords:** Prevention, Alcohol, Cigarettes, Marijuana, Dispositions, Skills, Mediating variables

## Abstract

The goal of this study was to evaluate a mediation model of two approaches to deterring the onset of alcohol, cigarette, and marijuana among middle school adolescents. Students completed surveys that included yes/no self-reports about their past 30-day and lifetime alcohol, cigarettes, and marijuana use. Surveys assessed *dispositions*: perceptions that drug use would interfere with desired lifestyles, perceptions about drug use prevalence and acceptability, and drug use intentions. Surveys also assessed *skills*: students’ ability to achieve goals, make decisions, and refuse drug use offers. Classrooms were assigned by convenience to one of three conditions. In the control condition, students (*N* = 394) received no prescribed intervention. *Core* condition students (*N* = 101) received instruction in *All Stars Core*, which targeted changing students’ dispositions. Students in the combined *Core and Plus* condition (*N* = 135) received instruction in both *All Stars Core* and in *All Stars Plus*, which also targeted improving students’ skills. Analysis revealed that the programs achieved reductions in the onset of drug use through maintaining these students’ dispositions. In contrast, control students’ dispositions eroded over time, increasing their risk for drug use. The *Plus* intervention that targeted skills, failed to have a sufficiently large impact to allow analysis to validate skills as a statistically significant mediator of drug use outcomes. These results speak to the conclusion that, for prevention programs to succeed at deterring the onset of alcohol and drug use, interventions need to maintain or improve students’ dispositions. This includes improving lifestyle incongruence, reducing perceptions that drug use is common and acceptable, and by increasing commitments to avoid drugs. Interventions that focus on building skills are less likely to achieve preventive effects.

## Introduction

Programs to deter the onset of alcohol, cigarette, and marijuana use now have more than four decades of research. Beginning with intervention studies in the mid-1970s through the early 1980 s (Evans et al., 1978; Goodstadt, 1975; Goodstadt et al., 1982; McAlister et al., 1979; Schaps et al., 1982), numerous strategies were developed and tested. Early interventions were based on a variety of social psychological concepts. In addition to providing students with information about licit and illicit drug use, these interventions included inoculating teens against peer pressure and peer influence (McGuire, 1964) and increasing self-efficacy for refusing drug use offers (Bandura, 1982), training teens to make thought out decisions and form no-use commitments (Janis & Mann, 1977), and combatting the false consensus effect by reducing estimated prevalence of use (Ross et al., 1977). Among the school-based programs that have received extensive research have been *Life Skills Training* (Botvin & Griffin, 2015) and D.A.R.E. (Evans & Tseloni, 2019; Ferreira-Junior et al., 2023; Pan & Bai, 2009; Sanchez et al., 2021).

In the succeeding decades, researchers focused on two priorities: documenting program effectiveness and examining predictors of substance use onset. These were initially considered separate foci as programs were created relying on the theoretical or conceptual understanding of the program developer and research in predictors of substance use focusing on developing strategies for testing relations between a variety of psychosocial variables and substance use.

### Documenting Program Effectiveness

By the mid-1980s, enough substance use prevention programs had been researched that it was possible to complete a meta-analysis examining intervention effectiveness (Tobler, 1986). In her meta-analysis, Tobler identified five broad categories of programs: knowledge only programs, affective only programs, peer programs (which included refusal skills training and social/life skills training), programs that included both knowledge and affective approaches, and alternative-focused programs (that provided positive activities or deficit compensation activities). The list of categories was subsequently appended to distinguish refusal skills (which included public commitments and cognitive behavioral skills) and generic skills (which included skills related to communication, assertiveness, decision making, problem solving, goal setting, and social skills) (Tobler et al., 2000). Comprehensive programs have been seen as most effective (Lemstra et al., 2010).

A separate review of school-based substance prevention programs specifically categorized the psychosocial variables targeted for intervention (Hansen, 1992). For each of 42 curricula, the inclusion of 12 variables were noted. Information was included in 90.5% of the programs, refusal skills were addressed in 45.2% of the interventions, decision-making skills were taught 40.5% of the time, followed by making commitments or pledges (33.3%), addressing social norms (28.6%), values (26.2%), teaching alternatives (23.8%), stress management (16.7%), self-esteem (16.7%), generic life skills (14.3%), goal setting (11.9%), and providing assistance (7.1%). Programs that addressed information, decision making, refusal skills, norm setting, and making commitments, categorized as comprehensive programs, overall had the most positive outcomes.

In the United States, which had been the setting for much of the research on substance use prevention prior to 2000, the Substance Abuse and Mental Health Services Administration (SAMHSA) was tasked with creating a database known initially as the *National Registry of Evidence-based Programs and Practices (NREPP)* (Brounstein et al., 2006). Over the next decade, numerous school-based programs qualified for inclusion in NREPP which began the process of widescale dissemination and the local adoption of programs. Much of the funding for dissemination was provided through grants from the US Department of Education and the Center for Substance Abuse Prevention (CSAP). (Brounstein et al., 2007; Elliott & Mihalic, 2004; Hogan et al., 2003).

More recently, there have been numerous meta-analyses and systematic reviews (Allara et al., 2015; Cho & Cho, 2021; Foxcroft et al., 1996; Hennessy & Tanner-Smith, 2015; Hodder et al., 2017; Lize et al., 2017; MacArthur et al., 2016; Pan & Bai, 2009) that have addressed the effectiveness of alcohol, tobacco, and marijuana programs. Most of these reviews focus attention on validating effectiveness with little attention given to how programs achieve effects.

### Modeling Mediating Variable Effects

Given the universal focus of prevention programs on targeting psychosocial variables, it became commonplace for studies that evaluated the effectiveness of programs to also measure and consider the role the variables played in understanding outcomes. The development of methods for assessing the paths through which interventions achieved their outcomes were soon widely applied. This involved mediating variable analysis (Judd & Kenny, 1981; MacKinnon, 1994, 2008; O’Rourke & MacKinnon, 2019), occasionally completed using structural equation modeling (SEM) (Bentler & Speckart, 1979; Jöreskog & Sörbom, 1982).

Among the first applications of mediating variable analysis was an examination of the outcomes of the Midwest Prevention Project (MacKinnon et al., 1991). The evaluation of the school-based intervention, *Project STAR* (Hansen et al., 1988; Pentz et al., 1989). The intervention consisted of ten sessions that addressed correcting erroneous normative perceptions, responding to peer pressure, assertiveness, problem solving, and making a public commitment to avoid substance use. Pretest and posttest surveys assessed alcohol, cigarette, and marijuana use as well as four psychosocial scales: friends’ reactions to use (injunctive normative beliefs), intentions, communication skills, and beliefs about the positive consequences of use. Only friends’ reaction to use, and intentions were found to mediate behavioral outcomes. Improvement in communication skills appeared to have an iatrogenic effect. Resistance skills, and negative consequences of drug use were not found to be significant mediators.

The *Adolescent Alcohol Prevention Trial (AAPT)* was a research project that evaluated two approaches to prevention (Hansen & Graham, 1991). One approach focused on correcting erroneously high normative beliefs about the prevalence and acceptability of substance use (descriptive and injunctive normative beliefs) while the second approach taught skills for resisting peer pressure to use substances. There were main effects for the normative education approach and no significant effects of resistance skill training. In a path analysis that examined how each intervention changed targeted mediators, resistance skill training improved resistance skills but had no significant path to any of the substance use measures. On the other hand, normative education reduced reported offers to use substances and improved students’ beliefs that substance use was unacceptable to the peer group. Paths between normative belief mediators and behaviors were significant (Donaldson et al., 1994).

*Life Skills Training* was developed as a school-based cigarette smoking prevention study in the early 1980s (Botvin et al., 1980) and subsequently expanded to include alcohol and marijuana use (Botvin, 2000). The program included five components: information about short-term consequences of use; information about the prevalence and acceptability of use; decision-making skills training; strategies for coping with anxiety; social and assertiveness skills training; and skills for self-improvement. The program significantly improved beliefs the acceptability of peer and adult smoking, beliefs about the prevalence of smoking, and knowledge of smoking consequences. A mediating variable analysis revealed that reductions in smoking prevalence were accounted for with beliefs about prevalence and acceptability (Botvin et al., 1992). Improvements in decision-making, coping, and social skills and assertiveness did not mediate program outcomes.

In addition to these landmark studies, numerous other mediating variable analysis studies have been conducted. Using data from an evaluation of *Project ALERT* (Orlando et al., 2005), structural equation models for program effects on cigarette- and alcohol-specific mediators were created. *Project ALERT* includes instruction that promotes self-efficacy to refuse offers, denigrates positive beliefs, and promotes negative beliefs about the consequences of use, and corrects normative perceptions that substance use is common and acceptable. For cigarettes, the intervention improved consequence and normative beliefs but had no effect on self-efficacy. In contrast, for alcohol, all four variables improved. While the successfully targeted variables mediated use, the larger impact observed was that each of these variables altered participants’ intentions to use.

*Project Northland* teaches students to deal with peer influence and corrects normative expectations about alcohol (Komro et al., 2001). The program achieved reductions in alcohol use by decreasing peer influences (perceived peer use and offers to use) and increasing the functional meaning of non-use.

The *Aban Aya Youth Project* (*AAYP*) (Liu et al., 2009) targeted behavioral intentions, self-efficacy, change in participants’ attitudes toward the behavior, estimates of peers’ behaviors, best friends’ behaviors, and peer group pressure. The intervention failed to change intentions and self-efficacy. Intervention effects on adolescent drug use were mediated by normative beliefs of prevalence estimates, friends’ drug use behavior, perceived friends’ encouragement to use, and attitudes toward the behavior.

Giannotta and her colleagues (Giannotta et al., 2014) examined the factors mediating the effects of a European school-based intervention, *Unplugged*. Program outcomes were mediated by improvements in attitudes toward drugs and refusal skills. For cigarettes, but not alcohol use, changes in normative beliefs also mediated outcomes. Several targeted mediators were changed by the program, but mediated paths were not completed because of weak relationships between the mediators and behaviors.

Several studies have examined *#Tamojunto* and *#Tamojunto2.0*, Brazilian versions of *Unplugged* (Giannotta et al., 2014). The initial version of the program, *#Tamojunto*, was found to either have no mediated effects (Sanchez et al., 2019) or a contraindicated indirect effect whereby #Tamojunto increased polydrug use by decreasing decision-making skills (Valente et al., 2020). The program was subsequently revised to improve its effectiveness. The revised program, *#Tamojunto2.0* included increased attention on changing normative beliefs (Sanchez et al., 2019). The revised intervention indirectly prevented lifetime alcohol use and binge drinking by increasing negative and non-positive alcohol beliefs (Garcia-Cerde et al., 2023). In a separate study of the Brazilian version of D.A.R.E., *Programa Educacional de Resistência às Drogas e à Violência (PROERD)*, mediating variable analysis found no effects of the psychosocial variables targeted by the program, communication skills, decision-making skills, attitudes toward drugs, and refusal skills (Valente & Sanchez, 2023).

Several recent systematic reviews have examined prevention program effectiveness (Egga et al., 2024; Medina-Martinez & Villanueva-Blasco, 2023; Mesas-Hueraman et al., 2023; Valente et al., 2022). Many of these reviews fail to find consistent positive outcomes. Nonetheless, it is notable that interventions that target correcting erroneous normative beliefs are among the few that achieve consistent positive effects (Lahiri et al., 2025).

### The Current Paper

The objective of this study was to examine the relative potential of a disposition-focused intervention versus a disposition plus skill intervention on changing the developmental patterns of targeted mediators and behavioral outcomes. In this paper, we will examine the effectiveness of two broadly defined approaches to prevention. The first approach (*All Stars Core*) involves a strategy to promote dispositional qualities that lessen the appeal of alcohol and drug use through strengthening personal commitments to avoid substance use, promoting non-use social norms, and bolstering perceptions that substance use is incongruent with lifestyle goals. The second approach (*All Stars Plus*) focuses on the development of personal and social skills: decision-making skills, goal-setting skills, and peer-pressure-refusal skills.

Prior research (Hansen & Dusenbury, 2004) assigned classrooms to one of three conditions: *All Stars Core*, *All Stars Core and All Stars Plus* combined, and a non-treated control. Results revealed that the combined *All Stars Core and All Stars Plus* program was more effective in preventing drug use than *All Stars Core* by itself. Both programs had lower rates of onset than did the non-treated controls. Initial findings suggested that rather than achieving the observed increase in effectiveness by changing targeted personal and social skills, the increase was due to a bolstered effect associated with increases in dispositional variables. However, analyses to confirm the latter observation was not completed. The goal of this paper is to examine the mediating effects of the two interventions.

In juxtaposition to the skills variables, in this paper we refer to lifestyle incongruence, intentions/commitment, and normative beliefs about peer drug use prevalence and acceptability as dispositional variables (Hansen & Hansen, 2024). Recent analysis of data from 25 pooled research projects reveals that the normal pattern of development is for dispositional variables to start with positive values that erode over time. The implication for prevention interventions is that, when addressing these variables, the goal of a program is to improve disposition where possible, but to primarily focus on maintaining positive disposition.

## Method

### Participants and Settings

Seventh grade classes were recruited to participate in the study. Students from eight schools participated in this study. Two schools were in South Carolina and six in Texas.

Of 770 students whose parents provided informed consent and who completed both pretests and posttests, survey data was available for 630 students. Of these, 52.3% were White, 30.6% were Black, 9.5% were Hispanic, 0.9% were Asian, 0.9% were Native American, 4.5% were “Other,” and 1.3% declined to answer. Females comprised 51.0% of the sample. Students ranged in age from 11 to 15 years with 49.5% being 12 years old and 42.7% reporting they were 13 years old at pretest with a mean age of 12.5 years. At posttest, the average age was 12.9 years with 23.6% then being 12 years old and 62.5% being 13 years old.

Additional demographic information includes the following. Texas schools were from rural areas with a total population of 3025 based on the US 2000 census. Median household income was $24,600 and 19% of families were below the poverty line. South Carolina schools were from a suburban area of 30,258 based on the census. Median household income from the 2000 census was $33,116 and 23% of the population was from families below the poverty line.

### Interventions

*All Stars Core* is a 14-session classroom program (Hansen, 1996; Harrington et al., 2001). The focus of the intervention is on three mediating variables: (1) improving students’ beliefs that drinking alcohol, getting drunk, smoking cigarettes, and using marijuana would interfere with their desired life goals; (2) correcting beliefs that exaggerate perceptions about the prevalence and acceptability of substance use among the peer group; and (3) encouraging students to make voluntary commitments to avoid substance use as minors.

*All Stars Plus* was designed to be a booster intervention to be delivered after completing *All Stars Core* (Hansen & Dusenbury, 2004). *All Stars Plus* added 11 teaching sessions that focused on: (1) teaching goal-setting skills with a reference in the instruction that linked goal setting with pursuing previously identified life goals; (2) teaching decision-making skills with a reference to aligning decision outcomes that maintained commitments students made during *All Stars Core*; and (3) teaching skills to refuse offers to engage in substance use with an emphasis that peer pressure should not be expected because of the existing low prevalence and the predominant unacceptability of substance use that had been defined in *All Stars Core*.

Both programs used interactive teaching pedagogies with discussions, small group activities, games, and role plays. In both programs, each session was designed to be delivered in a 45-minute class period.

### Research Design

Assignment to conditions was not randomized but rather was based on convenience. Students in South Carolina received the program in their health class. Two health teachers delivered the program. One taught *All Stars Core*, the other taught *All Stars Core* followed by *All Stars Plus*. Both teachers were trained in-person by the research team. Both attended the same *All Stars Core* training. The *All Stars Plus* teacher receive individual training on concepts and methods for teaching that curriculum. Students in South Carolina schools who were not enrolled in health served as a within-school controls. Students in an additional school in South Carolina served as a control site.

In Texas, a teacher who had taught *All Stars Core* in the past taught both *All Stars Core* and *All Stars Plus* over the course of two semesters. She was recruited because of multiple years of implementation and her enthusiasm for the program. She received *All Stars Core* training by attending an in-person training. She received *All Stars Plus* training via phone. Two schools received *All Stars Core* and *All Stars Plus* and three schools in Texas received only *All Stars Core*. One school served as a control group.

We employed no cross-contamination suppression measures, per se. Interventions were specific to schools. That is, All Stars Core and the combined All Stars Core and Plus curricula were always taught in different schools. In Texas control schools received no program. In South Carolina, program were delivered in health education classes. Control classes were on a schedule during which students participated in an alternative course of study other than health education.

For schools assigned to delivery of the program, student pretest surveys were administered before the program. Students in Texas were pretested in October. Students in South Carolina were pretested in January. Students in both states were posttested in May. For the current analysis, 394 students were available as controls, 101 received only *All Stars Core*, and 135 students received both *All Stars Core* and *All Stars Plus*.

### Measures

Student surveys assessed demographic information (age in years and gender). Students self-reported their lifetime and past 30-day Alcohol, Cigarette, and Marijuana use. Self-report measures were dichotomous (yes/no). A positive self-report about use for any of the three substances was used to calculate Any Use.

Six mediating variables were assessed. The three variables targeted by *All Stars Core* included Lifestyle Incongruence (5 items; α = 0.772), Normative Beliefs (8 items; α = 0.828), and Commitment to Avoid Substance Use (8 items; α = 0.856). The average of these variables was used to create a summary variable called “Disposition.” The three mediators targeted by *All Stars Plus* included Goal-Setting Skill (6 items; α = 0.794), Decision-Making Skill (11 items; α = 0.697), and Refusal Skill (9 items; α = 0.860). The average of these variables was used to create a summary variable called “Skill.” All measures were formatted so that responses ranged from 0 to 10 with higher values representing more theoretically desirable outcomes.

### Basis for Hypothesis Development

Understanding mediating variables is logically central to forming hypotheses about the possible effectiveness of an intervention. The law of maximum expected potential effect (Hansen & McNeal, 1996) summarizes the statical theorem that addresses the degree to which interventions that target psychosocial mediating variables that are designed to change behavioral outcomes can be effective. Among the basic assumptions of mediating variable analysis is that there are two requirements for a mediating effect to be determined. First, the intervention must have a significant impact on a targeted mediator. This can be achieved either because mediators in the treatment condition improve or because mediators in the control condition worsen. Second, there must be a significant path between the mediator and the program’s targeted outcome. Significance is required for both.

Eq. 1 reveals that the ultimate potential effectiveness of an intervention ($\:{ES}_{b}$) relies on how well the intervention changes the targeted mediator ($\:{ES}_{m}$) and the strength of relationship between the mediator and the behavior ($\:{\beta\:}_{mb}$). The size of effects can be odds ratios or other indicators of the magnitude of effect.

1 $$ES_{b} = \frac{{ES_{m} \times \beta _{{mb}} }}{{\sqrt {\frac{{\sqrt {(ES_{m}^{2} \times se\beta _{{mb}}^{2} ) + (\beta _{{mb}}^{2} \times seES_{m}^{2} } )}}{{N_{1} + N_{2} - 2}}} }}$$

An analysis of data from 25 pooled projects (publicly available at: 10.5281/zenodo.5256140) for which skill variables had been assessed (Hansen et al., 2022) revealed that, on average, correlations between these variables and self-reported alcohol, cigarette, and marijuana use were relatively weak. For example, the average correlation between goal-setting skill and alcohol, cigarette, and marijuana use was 0.171. Average correlations with decision-making skill (*r* =0.140) and refusal skill (*r* =0.181) were within the same range. In contrast, an examination of disposition variables in the same dataset revealed that intentions to not use (*r* =0.506), perceived incongruence between one’s desire lifestyle and drug use (*r* =0.322), and perceived peer norms (*r* =0.283) were strong predictors of use.

**Fig. 1:**
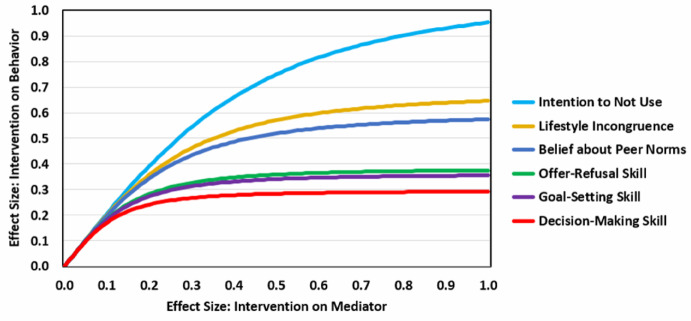
Potential of six targeted mediators to achieve changes in behavior

Applying Eq. 1 to these results yields the potential for program effective size shown in Fig 1. In this figure, changing intentions to not use (commitments to avoid use) has a clearly superior potential for altering drug use outcomes. Beliefs about lifestyle incongruence and peer norms also hold promise for affecting outcomes. In contrast, each of the skill variables have less preventive potential. Even with a large effect on improving goal-setting, decision-making, and refusal skills, can only have small effects on behavioral outcomes.

### Hypotheses and Analysis Plan

**Fig. 2:**
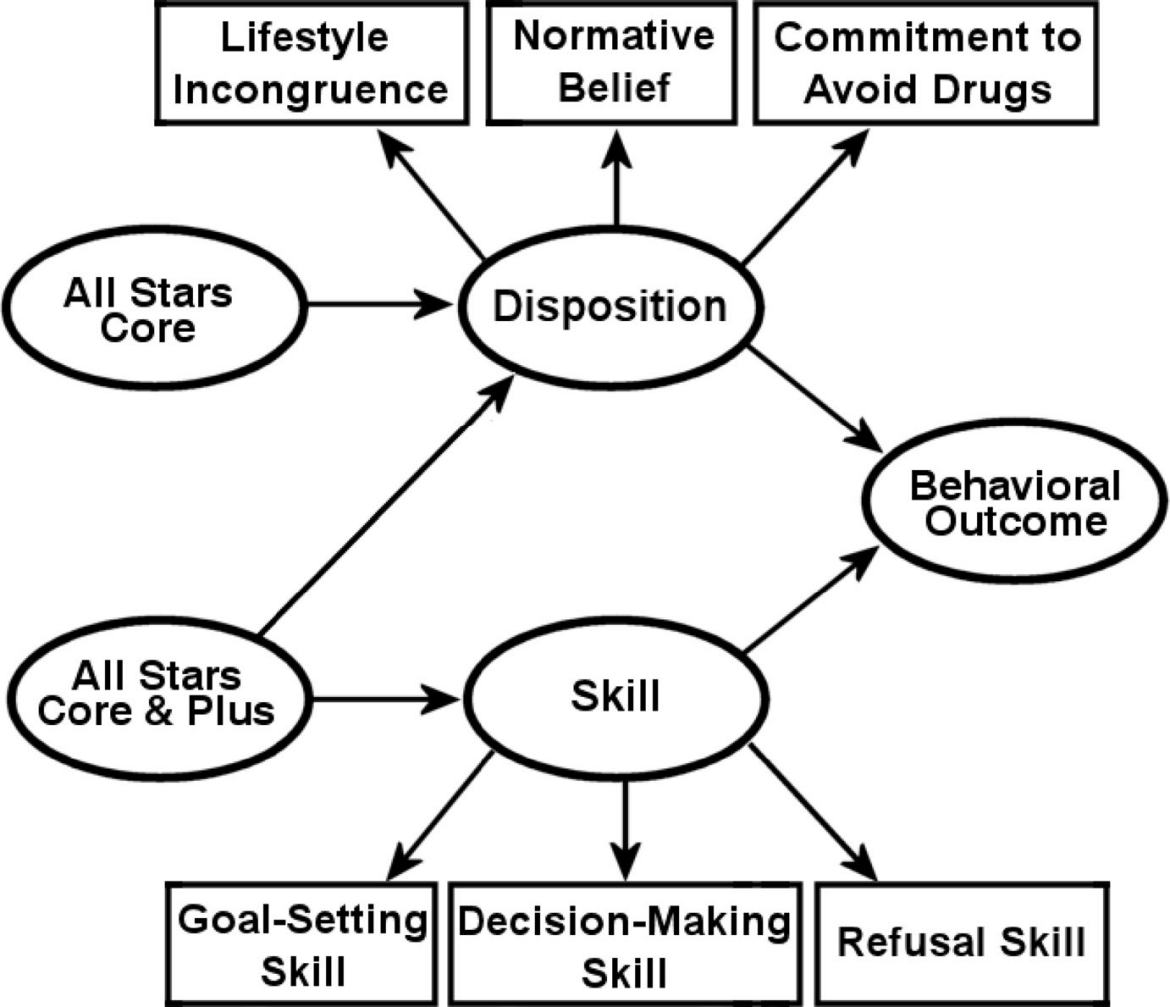
Hypothesized mediating variable paths

Figure 2 presents the model for hypothesized mediating effects. The *All Stars Core* program targets the three mediators that reflect a student’s disposition. We hypothesized that if *All Stars Core *was successful as a disposition-focused intervention, the result would be maintaining students’ Disposition scores (Hansen & Hansen, 2024). In contrast, the control group’s Disposition would be comparatively lower from pretest to posttest. This would result in treatment student’s likelihood of using drugs to remain low compared to the control group. To the extent that *All Stars Plus* also focuses on improving or maintaining a positive disposition, we similarly hypothesized that, compared to the control group, students in the combined *All Stars Core and Plus* condition would have a similar positive effect on Disposition and subsequently on deterring the onset of drug use

The model also predicts that, due to the instruction provided about goal setting, decision making, and refusing offers to use drugs, students in the combined *All Stars Core and Plus* condition would have increased Skill at posttest compared to students in the control condition. The normal trend in skill development is not known, although ideally the intervention would improve students’ self-reported Skill. Any improvements in students’ skill would theoretically lower the student’s chances of using drugs, although as noted in the discussion about potential effectiveness of these mediating variables, their impact on behavior is expected to be smaller than the impact of the program via changes in disposition.

Planned analyses consist of a series of binary logistic regression equations for 30-day and lifetime usage of Alcohol, Cigarettes, Marijuana, and Any Use. For these analyses, we use cluster robust standard errors (CRSEs) to account for the clustering of students within schools to reduce the Type I error. As they are dichotomous measures, we rely on using a macro within SPSS developed by Huang and Li (2022) specifically developed to account for clustering within a small number of clusters when examining dichotomous outcomes. To conduct the mediation analysis, we first examine linear regression estimates for the mediator variables; this analysis also corrects the standard errors for clustering within schools and relies on the PROCESS macro V5.0 (Hayes, 2022).

To assess the statistical significance of any indirect effects, we used the SPSS program using the PROCESS macro (Hayes, 2022). Traditionally, statistically significant indirect effects could be determined using the Sobel method (Sobel, 1982), but for that method to be appropriate the distribution of the standard errors for the indirect effects must be normally distributed. Given our outcome variables are dichotomous and highly skewed, the standard errors for the indirect effects do not follow a normal distribution. As such, we have followed other researchers and have used a bootstrapping sub-sample technique, with replacement. We used 10,000 sub-samples to create a distribution for the indirect effect standard errors and present the lower and upper estimates that correspond to the 95% confidence interval as a guide for statistical significance.

To help with interpreting the estimated chances of adolescents using drugs, gender was recoded as 1 for males and 0 for females. Age was originally coded as years old at wave 1 and ranged from 11 to 15 years old; for the multivariate analyses, we have recoded age from 0 to 5 with 0 being 11 years old and 5 being 15 years old. This does not affect the covariate structure for the analysis but does help with interpretation as the intercept now is the average likelihood of 11-year-old females using particular drugs. The gender coefficient is then the average difference between males and females; the age coefficient is the estimated change in the likelihood of using drugs for each additional year of age in the sample.

## Results

Table 1 Presents condition-by-condition and pretest-posttest descriptive statistics. For both disposition and skill, there were notable declines between pretest and posttest for the control group (−0.11, and −0.08, respectively). students who received the *Core* program slightly increased their average disposition score (+0.02) but had their skill score moderately decrease (−0.02) students who received the combined *Core and plus* program had a slight increase in their average disposition score (+0.01) and a pretest-posttest increase in their skill score (+0.01).

Control group pretest-posttest decreases were observed for all three sub-class measures: −0.07 for Lifestyle Incongruence and −0.11 for Normative Beliefs and Commitment to Avoid Drug Use. Students in the *Core* and the combined *Core and Plus* conditions had improved Lifestyle Incongruence scores (+0.04 and +0.02, respectively). They had worsening Normative Beliefs scores (−0.06 and −0.01, respectively). Students in the *Core* condition improved their Commitment to Avoid Drug Use scores (+0.05) whereas those in the combined *Core and Plus* condition had no pretest-posttest change.

**Table 1:** Characteristics by Condition and Testing Period

		Control		Core		Core and Plus	
		Pretest	Posttest	Pretest	Posttest	Pretest	Posttest
Psychosocial Variables: Means and Standard Deviations*							
	Disposition	7.96 (0.56)	7.85 (0.65)	7.92 (0.60)	7.94 (0.61)	8.04 (0.50)	8.05 (0.50)
	LI	8.18 (0.60)	8.11 (0.72)	8.20 (0.59)	8.24 (0.65)	8.23 (0.63)	8.25 (0.63)
	NB	7.02 (0.65)	6.91 (0.66)	6.91 (0.76)	6.85 (0.70)	7.06 (0.62)	7.05 (0.62)
	C	8.68 (0.69)	8.57 (0.79)	8.66 (0.77)	8.71 (0.75)	8.84 (0.58)	8.84 (0.61)
	Skill	6.99 (0.59)	6.91 (0.67)	7.02 (0.61)	7.00 (0.54)	6.97 (0.51)	6.98 (0.53)
	GS	6.74 (0.71)	6.59 (0.74)	6.75 (0.71)	6.78 (0.69)	6.69 (0.65)	6.69 (0.71)
	DM	5.51 (0.77)	5.35 (0.85)	5.58 (0.76)	5.40 (0.69)	5.43 (0.68)	5.36 (0.74)
	R	8.65 (0.70)	8.71 (0.73)	8.75 (0.79)	8.79 (0.71)	8.80 (0.65)	8.85 (0.59)
*Behaviors: Percent Reporting Use*							
	Alcohol 30-Day	9%	10%	8%	3%	6%	3%
	Alcohol ever	28%	38%	28%	39%	27%	31%
	Cigarette 30-Day	7%	9%	11%	12%	3%	2%
	Cigarette ever	24%	30%	30%	35%	16%	24%
	Marijuana 30-Day	3%	5%	5%	4%	1%	0%
	Marijuana ever	5%	8%	9%	14%	2%	4%
	Any use 30-Day	23%	22%	17%	21%	16%	7%
	Any Use Ever	48%	57%	42%	55%	33%	39%

* LI =Lifestyle Incongruence, NB = Normative Beliefs, C = Commitment to Avoid Drug Use, GS = Goal Setting Skill, DM = Decision Making Skill, and R = Refusal Skill

Reported Goal-Setting Skill and Decision-Making Skill among control students became worse from pretest to posttest (−0.15 and −0.16, respectively). Whereas students who participated in the *Core* program reported an improvement in their Goal-Setting Skill (+0.03), students in the *Core and Plus* condition reported no pretest-posttest change. Decision-Making Skill decreased for students in both the *Core* and the combined *Core and Plus* conditions (−0.18 and −0.07, respectively). In contrast, students in all three conditions (control, *Core* and *Core and Plus*) reported improvements in Refusal Skills (+0.06, +0.04, and +0.05, respectively).

Pretest self-reported alcohol use was consistent among the conditions. However, the prevalence of smoking varied considerably with student in the Core condition having more experience with smoking cigarettes at pretest than students in the other conditions. The prevalence of marijuana use at pretest was low among all conditions. The pretest Any Use variable varied among the conditions. Control condition students had more pretest prevalence of Any Use, both past 30-day and lifetime.

Mediating variable analysis rests on understanding the relationship between the targeted mediator and the behavior of interest. Table 2 presents the pretest correlations between self-reported use and the two targeted mediators included in the hypothesized model. Average correlations for Disposition were stronger than those associated with Skill.

**Table 2:** Bivariate Correlations between Pretest Use and Targeted Mediators

	Disposition	Skill
Alcohol 30-Day	−0.377	−0.204
Alcohol ever	−0.514	−0.355
Cigarette 30-Day	−0.462	−0.294
Cigarette ever	−0.528	−0.341
Marijuana 30-Day	−0.318	−0.148
Marijuana ever	−0.430	−0.214
Average	−0.438	−0.259

 Table 3 Includes the log-odds estimates from a binary logistic regression of drinking alcohol, smoking cigarettes, and using marijuana in the past 30 days. It also includes the log-odds estimates of having used any of the drugs in the past 30 days. All statistically significant results are determined using the cluster robust standard errors (CRSEs) to account for the clustering of students within schools. When comparing the statistical significance of the results, disposition significantly reduced the likelihood of using drugs whereas skill did not. In terms of magnitude, a 1 unit increase in disposition is associated with significantly lower estimated odds of 30-day alcohol (exp −0.719, OR = 0.49), 30 day cigarette (exp −0.717, OR = 0.49), 30-day marijuana (exp −0.985, OR = 0.37) and 30-day any use (exp −0.723, OR = 0.49).

**Table 3:** Log-Odds of Past 30-Day Drug Use (CRSEs)

	Alcohol		Cigarettes	Marijuana	Any Use
Intercept	2.913**		3.316*	2.152	5.387**
Gender	0.367		0.715**	1.210**	0.322
Age	0.051		−0.071	0.229	−0.293
White	0.423		0.624**	−0.253	0.155
Posttest disposition	−0.719**		−0.717**	-.985*	−0.723**
Posttest skill	0.084		−0.148	0.422	0.095
Core	−2.384**		1.243**	−1.397*	0.295*
Core and Plus	−0.805*		−2.191**	−17.447**	−1.223**
−2 Log-Likelihood	192.4		132.9	91.8	335.6

All models control for Pretest use and Pretest disposition and Skill.

* < 0.05; ** < 0.01

Results also indicate that being in the *Core* program group was associated with a significant direct reduction in 30-day alcohol use and marijuana-se, but a significant increase in the likelihood of past-month smoking cigarettes or using any drugs. Those students who received both the *Core* and *Plus* programs had lower likelihoods of using any form of drugs within the 30-day window of the posttest, even after controlling for pretest drug usage, dispositions, and skills. Table 4 Presents results for reporting ever having used the listed drug at posttest; the statistical analyses also account for the clustering of students within schools. It is a near replica of table regarding disposition’s consistent ability to help reduce the estimated likelihood of using drugs. In terms of magnitude, a 1 unit increase in disposition is associated with significantly lower estimated odds of reporting ever using alcohol (exp −0.377, OR = 0.69), cigarettes (exp −0.490, OR = 0.61), marijuana (exp −0.912, OR = 0.40) or any drug (exp −0.494, OR = 0.61). The *Core* program group reported significantly higher odds of ever using marijuana. There are no statistically significant direct effects between the combined *Core and plus* program groups and the control group in posttest drug use once disposition and skill are statistically controlled for.

**Table 4:** Log-Odds of Ever Drug Use (CRSEs)

	Alcohol		Cigarettes	Marijuana	Any use
Intercept	2.631**		0.200	2.600	3.297**
Gender	0.075		0.071	0.693	0.187
Age	−0.055		0.269	0.063	0.108
White	0.304		0.153	−0.133	0.311
Posttest disposition	−0.377*		−0.490**	−0.912**	−0.494**
Posttest skill	0.025		−0.255**	0.189	−0.032
Core	0.127		0.087	0.813*	0.278
Core and Plus	−0.301		0.220	0.151	−0.312
−2 Log-Likelihood	505.1		348.5	157.7	460.7

All models control for Pretest use and Pretest disposition and Skill.

* < 0.05; ** < 0.01

Because *Core* was originally designed to focus on Disposition, and *Plus* was meant to increase Skill, Table 5 presents the linear regression models assessing each program’s ability to affect Disposition and Skill. In the clustered analyses, the *Core* program significantly predicted changes in Disposition. Neither program predicted changes in Skill.

Given that skills were only minimally statistically significant in predicting lifetime ever-use of cigarettes, and that neither program altered skills in any meaningful way, we pruned skills from the mediating variable analysis. Likewise, we present only the mediating analysis for 30 day drug use given Disposition’ consistent effect at reducing both 30-day and ever drug use measures.

**Table 5:** Linear Regression Estimates (Not Standardized) on Posttest Mediator Variables (CRSEs)

	Posttest Disposition	Posttest Skill
Intercept	2.412**	2.346**
Gender	−0.139**	−0.109
Age	−0.240*	−0.173**
White	−0.072	−0.011
Core	0.254*	0.156
Core and Plus	0.208	0.118
F Statistic (CR)	4740.374**	1849.661**
R-Square	0.522	0.442

The Posttest Disposition model controls for Pretest Disposition.

The Posttest Skill model controls for Pretest Skill.

* < 0.05; ** < 0.01

The mediating analysis presented in Table 6 lists the estimated program direct and indirect effects on 30-day drug use. The 95% lower and upper limits of the confidence interval from the 10,000 bootstrap estimates are noted in parentheses below the indirect effects. While not a formal statistical test, the common practice for interpreting results is that when the 95% confidence interval does not contain 0, then it is reasonable to conclude statistical significance has been achieved.

**Table 6:** Program Direct and Indirect Effects via Disposition, on Month Drug Use: Log-Odds

	Alcohol	Cigarettes	Marijuana	Any use
Core direct	−2.414**	1.198*	−0.975	0.297
Core indirect	**−0.153**	**−0.235**	**−0.177**	**−0.160**
(lower/upper CI)	**(−0.363, −0.003)**	**(−0.526, −0.044)**	**(−0.525, −0.001)**	**(−0.352, −0.002)**
Core and Plus direct	−0.820	−2.193	−14.398	−1.216**
Core and Plus indirect	−0.138	−0.176	−0.151	−0.147
(lower/upper CI)	(−0.344, 0.007)	(−0.451, 0.008)	(−0.473, 0.014)	(−0.323, 0.004)

All models control for Gender, Age, Race, Pretest use and Pretest disposition

* < 0.05; ** < 0.01; a bolded indirect effect indicates significance

In terms of direct effects, *Core* significantly reduces the likelihood of previous 30-day alcohol use and increases 30-day cigarette use. The combined *Core and Plus* program significantly reduced the likelihood of using any drug in the previous 30 days. The crucial finding is the degree to which the combined *All Stars Core* program significantly reduced the likelihood of 30 day drug use by altering a student’s Disposition. The Disposition mediated path (notated as “Core Indirect”) accounted for reductions in the odds of past 30 day Alcohol (exp −0.153 OR = 0.86), Cigarette (exp −0.235; OR = 0.79), Marijuana (exp −0.177; OR = 0.84) and Any Use (exp −0.160; OR = 0.85). The combined *All Stars Core and Plus* outcomes were similarly mediated by lowering the odds of 30-day Any Use (exp −0.147; OR = 0.86) via the pretest-posttest change in students’ Disposition.

## Discussion

For a targeted psychosocial variable to mediate an intervention’s outcome requires that there be both a difference in the mediator produced by the intervention and a strong relationship between the mediator and the intended outcome. The significance of the indirect effects can be attributed to both the relative declines in control students’ Dispositions and the strength of relationship between Disposition and various forms of drug use. In the case of prevention programs, it is the case that preventive effects are typically only observed when the control or comparison group worsens. Observed improvements in the intervention group is rare; it is much more likely that success is achieved when the status quo is maintained and there is continued non-use among students in the program condition.

Both *Core* and *Core and Plus* programs produced desired effects on students’ Disposition. While the magnitude of change in students’ reported Disposition scores was small, these changes nonetheless accounted for a preventive effect for students’ use of their recent alcohol, cigarette, marijuana, and poly-drug use and their lifetime alcohol and poly-drug use. The mediated impact observed was due to the strong correlation between Disposition and self-reported pretest drug use.

The *Plus* portion of the combined *Core and Plus* intervention did not specifically address additional curriculum components to reinforce students’ perceptions that drug use would interfere with their desired lifestyles, their perceptions that drug use was less prevalent or more unacceptable to the peer group, or to ask them to increase their commitments to avoid using drugs. The program only reinforced these concepts by noting to students that they had previously come to these intended outcomes as part of laying the foundation for pursuing instruction in how to set and achieve goals, how to make well-thought-out decisions, and how to respond should they encounter unwanted peer pressure. However, it is possible that the messages intended as part of *Core* were nonetheless functionally reinforced during the *Plus* portion of the intervention.

Compared to the control condition, the combined *Core and Plus* intervention condition had slightly better outcomes for improving students’ Skill. However, the pretest-posttest comparative improvement was modest when comparing these two conditions. Furthermore, because the correlations between drug use and the targeted mediator were also modest, no statistically significant indirect effects were observed.

We can only speculate about the failure of the skill-focused intervention to affect positive outcomes in students’ self-reported decision-making, goal-setting, and refusal skills. Many prevention programs include skills training as part of the primary focus of intervention. It is not unusual for programs to address skill development briefly, including only one or two lessons at most. While All Stars Plus included only two lessons that focused on goal-setting skills, the intervention gave increased attention to decision-making and refusal skills. There are several explanations for why skills were not significantly improved. For example, it may be that the intervention was not correctly designed. However, it is also possible that these skills are simply challenging to improve. They may require an extended period of instruction and extensive guided practice before positive changes can be observed.

### Limitations

Other tests comparing skills and dispositional approaches are clearly warranted. The current study was designed as a short-term pilot project. Since the time in the early 2000 s when this project was completed, there may have been many refinements to school-based curricula that could be included in longitudinal research studies that may have the potential to further demonstrate the relative benefits of skills and dispositional interventions. Research cited in the introduction that highlights the general trend for disposition-relevant mediators to account for programmatic outcomes, while skill-centered interventions have failed to do so, suggests that future research should attempt to tease apart these two lines of intervention.

The design of this evaluation was not a fully factorial. In an ideal test of skill versus disposition, a skill-only version (*Plus* by itself) would be included as a condition. Given the limited budget and the intent to include *Plus* as a booster program that keyed off the psychosocial changes that *Core* was intended to change, this was not possible.

Because the assignment to conditions was not randomized, it may have introduced bias into the results. Without random assignment, there is a possibility that selection bias may have played a role in observed outcomes. Testing for pretest non-equivalence, none was found; however, there may always be non-measured differences that were not observed. In the era of the dissemination of evidence-based programs, it is becoming more difficult to randomly assign schools and classrooms to control conditions. Nonetheless, a strategy for estimating the effects of untreated students should be pursued. Understanding the mechanisms through which interventions achieve or fail to achieve their outcomes remains an important research issue to pursue.

A final limitation is that the indirect effect analysis cannot fully account for the clustering of students within schools. We account for the possibility of Type I error using Cluster Robust Standard Errors in the direct analyses. However, the closest we can come to determining statistical significance for the indirect effects is the bootstrapping approach given the dichotomous dependent variables and the inability to use the Sobel (1982) test.

### Conclusions

Programs designed to deter the onset of alcohol, cigarette, marijuana, and other drugs are typically designed to target specific psychosocial mediators. Programs can achieve preventive outcomes when two conditions are satisfied. First, programs must target for change those psychosocial variables that have a strong statistical relationship with behavior. Prior research has demonstrated that dispositional variables (lifestyle incongruence, normative beliefs, and intentions to avoid drug use, as well as attitudes and beliefs) have a greater potential to qualify as suitable targets for intervention.

Second, interventions must change to course of development of targeted mediators. This is most often accomplished by maintaining pretest levels of targeted variables. This is particularly true for the variables described in this paper as those that are related to students’ dispositions. Dispositional variables typically erode as young people grow older. Thus, it is the negative changes in the control group following a natural, untreated course of decline that allows preventive effects to be achieved.
